# Editorial: Highlights in Lipids in Cardiovascular Disease: 2021

**DOI:** 10.3389/fcvm.2022.915262

**Published:** 2022-05-06

**Authors:** Chaymae Boucheniata, Nolwenn Tessier, Catherine Martel

**Affiliations:** ^1^Department of Medicine, Faculty of Medicine, Université de Montréal, Montreal, QC, Canada; ^2^Research Center, Montreal Heart Institute, Montreal, QC, Canada

**Keywords:** hyperlipidemia, hypertriglyceridemia, plasmapheresis, lipid metabolism, remnant cholesterol, non-statin cholesterol lowering drugs

This collection highlights a selection of articles published in 2021 from the Lipids in Cardiovascular Disease section of Frontiers in Cardiovascular Medicine. Lipids such as cholesterol and triglycerides (TG) are key contributors to cardiovascular disease (CVD) (1). They are transported in association with proteins in the circulation. These so-formed lipoproteins are complex particles divided into several classes based on their size, apolipoprotein and lipid composition. Chylomicrons, chylomicron remnants, VLDL, IDL, LDL, HDL, and Lp (a) have a central core containing cholesterol esters and triglycerides surrounded by free cholesterol, phospholipids and apolipoproteins (2). In 1961, the epidemiological Framingham Study demonstrated the association between high blood cholesterol levels and CVD (3). The subsequent “cholesterol hypothesis” that raised proposed that LDL-cholesterol (LDL-C) is instrumental for the development of atherosclerosis, the main underlying cause of CVD. Specific classes of lipoproteins thus began to be identified as triggers in the inflammatory processes, thereby promoting blood vessel inflammation and cardiomyopathies (4).

Lowering of LDL-C had become a target of interest in the reduction of the risk of myocardial infarction and other cardiovascular events (5). Hydroxymethylglutaryl-CoA reductase (HMG-CoA) reductase inhibitors, frequently referred to as “statins,” are the gold standard for the management of LDL-C. However, many patients develop adverse drug effects (ADEs) and are unable to tolerate cholesterol-lowering medication. This highlights the need for the development of drugs designed to target other lipid mediators beyond LDL-C in patients with statin intolerances or in whom a statin alone does not lower LDL-C sufficiently, for instance (6, 7). The 2018 American College of Cardiology (ACC) and American Heart Association (AHA) guideline indicated that the non-statin therapies of choice should be ezetimibe, bile acid sequestrants/resins, and PCSK9 inhibitors (8). In the first paper of this special issue, Bardolia et al. provide an insightful narrative review of non-statin therapies that are shown promising in reducing LDL-C, either as monotherapy or in combination therapy with statins or other non-statin medications (9). They emphasize on the pharmacokinetic, efficacy and safety profiles of drugs that either selectively inhibit cholesterol absorption by the intestine (e.g., ezetimibe), prevent *de novo* cholesterol synthesis in hepatocytes (e.g., bempedoic acid, BDA), or reduce proprotein convertase subtilisin/kexin type 9 (PCSK9) function by preventing its binding to the LDL receptors (e.g., alirocumab, evolocumab) or by inhibiting its production by the liver (e.g., inclisiran).

Beside non-statin therapies, other therapeutic options have emerged to meet the need for reducing circulating LDL-C. Double-filtration plasmapheresis (DFPP) is a low-cost treatment used to decrease LDL-C concentrations in patients with dyslipidaemias, such as homozygous familial hypercholesterolemia (HoFH) (10). In their original publication, Zhang et al. sought to investigate the potential effects of non-drug therapy with double-filtration plasmapheresis (DFPP) on lipid metabolism-, endoplasmic reticulum (ER) stress-, and apoptosis-related proteins in peripheral blood mononuclear cells (PBMCs) before and after lipid clearance in patients with hyperlipidemia (11). In line with a previous study that reported that lipid plasmapheresis reduced plasma PCSK9 levels (12), they show that DFPP induces the downregulation of PCSK9, CD36 and LDLR in PBMCs. In addition, the group reports that DFPP reduces the levels of ER stress- and apoptosis-related proteins and reactive oxygen species (ROS) in PBMCs.

Recent clinical trial evidence led to the reprioritization of the causal lipids responsible for the onset and progression of atherosclerotic cardiovascular disease (13). Hypertriglyceridemia (HTG) is a frequent form of dyslipidemia (14). TG-rich lipoproteins (TRL) and their remnants are now known as important contributors to atherosclerosis, the main underlying cause of CVD (15). TG can be either exogenous (transported in intestinally derived chylomicrons) or endogenous (circulating in hepatically-derived VLDL). In a retrospective study involving 12,563 patients, Kexin et al. (16) sought to evaluate the association between RC and non-HDL-cholesterol (HDL-C) with the risk of coronary artery disease (CAD), as diagnosed according to the 2019 guideline of the European Society of Cardiology (ESC) (17). Their overarching hypothesis was that RC is more capable to predict the risk of CAD than LDL-C and non-HDL-C. They have estimated non-HDL-C as total cholesterol minus HDL-C while RC was calculated as total cholesterol minus LDL-C minus HDL-C. Albeit several limitations have been acknowledged by the authors including the underlying mechanisms involved, this study reports a significant association between RC and CAD, and shows a correlation between RC and age, gender, hypertension, and diabetes in CAD progression.

Whereas, measuring TG levels provide a first approximation of the total circulating TRL and their remnants cholesterol (RC), there is no simple, widely available assay to measure the cholesterol content of TG-rich lipoproteins and remnants (18). Plasma TG levels are particularly influenced by the dietary intake. Guo et al. underscore that there is no consensus on the optimal cutoff value after a daily meal in the diagnosis of HTG in Chinese subjects. In their original article, they thus sought to determine the non-fasting cutoff value that corresponds to the target fasting level of 2.3 mmol/L in Chinese patients. From March 2017 to July 2020, they enrolled a cohort of 602 Chinese patients, including 120 with HTG (TG ≥ 2.3 mmol/L before admission). Blood lipid levels were measured at 0, 2, and 4 h after breakfast. The group reported that the levels of non-fasting TG increased significantly in both HTG and non-HTG subjects, and reached a peak at 4 h post-prandial. ROC curve analysis revealed that the optimal cutoff value used to predict HTG is 2.66 mmol/L, which brings the incidence of non-fasting HTG close to its fasting level (19). As a significant proportion of the patients included in this study were taking lipid-lowering agents, the authors underscore the need to carry out similar research on outpatients who do not receive lipid-lowering drugs.

The regulation of lipid metabolism can be the underlying cause of several cardiomyopathy. The A Disintegrin and Metalloprotease 17 (ADAM17) is a key regulator of inflammation and lipid metabolism in a specific cardiomyopathy, the Takotsubo cardiomyopathy (TTC). This disease consists of an acute, stress-induced cardiac syndrome characterized by a transient wall motion abnormality of the left ventricle (20). In their review paper, Adu-Amankwaah et al. underline that ADAM17 cleaves pro-inflammatory cytokines such as tumor necrosis factor α and interleukin 6 and activates nuclear factor kappa-light-chain-enhancer of activated B cells (NF-κB) signaling pathways (21). They suggest that there could be a strong correlation between the modulation of acute myocardial inflammation and metabolic lipids dysregulation by sex hormones and the endocrine system in TTC.

Altogether, original articles and reviews included in the 2021 Lipids in Cardiovascular Disease section of Frontiers in Cardiovascular Medicine gather new pathophysiologic insights onto the contribution and management of lipids in CVD.

## Author Contributions

CB wrote the first draft of the manuscript. NT translated the manuscript. CM corrected, edited, and finalized the manuscript. All authors listed approved the work for publication.

## Funding

This work was funded by the Canadian Institutes for Health Research Canada Research Chair (950-232250) and the Montreal Heart Institute Foundation to CM.

## Conflict of Interest

The authors declare that the research was conducted in the absence of any commercial or financial relationships that could be construed as a potential conflict of interest. The handling editor MS-T declared a past co-authorship with the authors.

## Publisher's Note

All claims expressed in this article are solely those of the authors and do not necessarily represent those of their affiliated organizations, or those of the publisher, the editors and the reviewers. Any product that may be evaluated in this article, or claim that may be made by its manufacturer, is not guaranteed or endorsed by the publisher.
